# Unusual association between enchondroma and Camurati-Engelmann disease: A case report

**DOI:** 10.3109/03009730903406777

**Published:** 2010-04-07

**Authors:** Hiroyuki Nagasawa, Kyoji Okada, Hiroshi Nanjo, Hiroshi Sasaki, Shuichi Chida, Yoichi Shimada

**Affiliations:** ^1^Department of Orthopedic Surgery, Akita University Graduate School of Medicine, AkitaJapan; ^2^Department of Clinical Pathology, Akita University Hospital, AkitaJapan

**Keywords:** Camurati-Engelmann, diaphyseal dysplasia, diagnosis, enchondroma, progressive

## Abstract

This case report describes an enchondroma of the distal phalanx of the right little finger in a 37-year-old woman with Camurati-Engelmann disease. Curettage of the tumor and artificial bone grafting were performed in May 2004. Surgical treatment resulted in a good clinical outcome with no evidence of recurrence at 5-year follow-up. The genetic relationships between Camurati-Engelmann disease and benign chondroid tumors are discussed.

## Introduction

Camurati-Engelmann disease (CED) is a rare genetic disorder with a prevalence of less than 1:10^6^ (1). Radiologically, CED is characterized by periosteal and endosteal sclerosis associated with thickening of the long bone diaphyses. Short tubular bones are affected less frequently. Enchondroma has been found to be associated with various skeletal dysplasias, including fibrous dysplasia, achondroplasia, and osteogenesis imperfecta (2). However, to our knowledge, an association between enchondroma and CED has not been reported previously. In this report, we describe the clinical, radiological, and histological findings of an enchondroma in the distal phalanx of the right little finger in a 37-year-old female with CED.

## Case report

**Figure 1.**
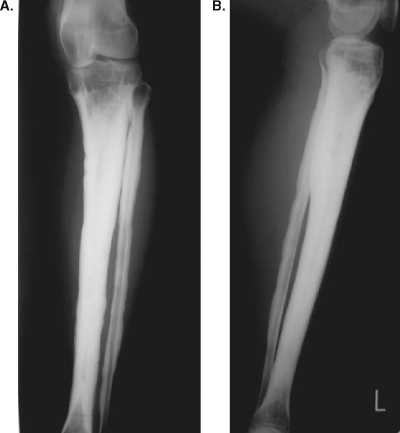
Anteroposterior (A) and lateral (B) radiograms of the left lower leg show diaphyseal widening and cortical thickening of the tibia and fibula.

In 1990, a 23-year-old Japanese woman initially presented to our hospital with upper and lower limb pain. Radiographs on admission showed diffuse, bilateral sclerosis of the medullary cavity of her femur and tibia along with endosteal and subperiosteal cortical thickening affecting the diaphysis with epiphyseal sparing (Figure 1). The patient was diagnosed as having progressive diaphyseal dysplasia (Camurati-Engelmann disease), which was treated with oral administration of non-steroidal anti-inflammatory drugs and low-dose prednisolone (8 mg/day).

**Figure 2.**
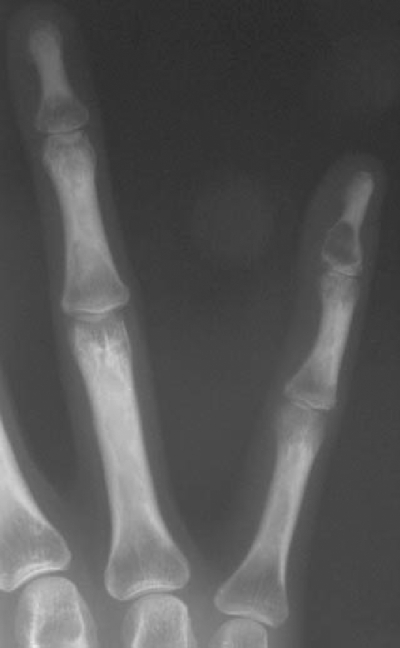
Anteroposterior radiogram of the distal phalanx of the little finger shows distension and thinning of the cortex around a radiolucent lesion.

In 2004, this patient was admitted to our institute at age 37 with a complaint of pain in the distal phalanx of her right little finger. Physical examination showed swelling and tenderness of the right little finger, despite which she had full range of motion in all fingers. Radiographs showed an osteolytic lesion in the distal phalanx of the little finger with distension and thinning of the surrounding cortex (Figure 2). The clinical diagnosis was enchondroma.

**Figure 3.**
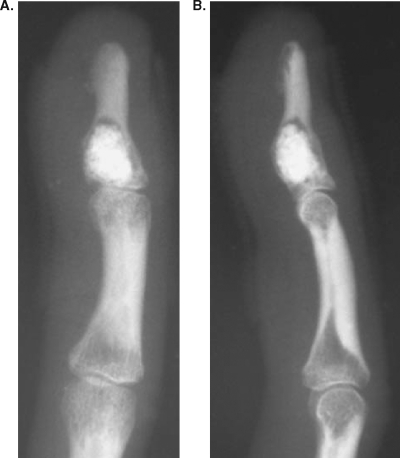
Post-surgery, anteroposterior (A) and lateral (B) radiograms of the little finger show artificial bone completely filling the cavity in the distal phalanx previously occupied by the tumor.

**Figure 4.**
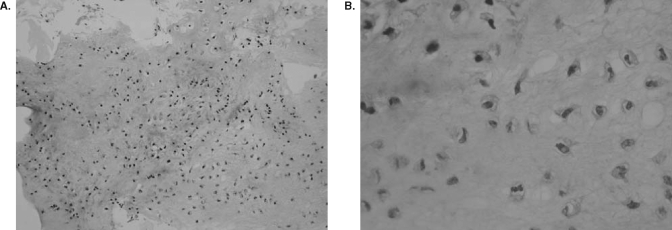
Photomicrographs of the tumor from the distal phalanx show chondroid matrix and no atypical tumor cells (A: ×100; B: ×200).

Surgery was performed in May 2004. A longitudinal skin incision was made on the dorsal ulnar aspect of the distal phalanx of the right little finger. A cortical hole was easily made in the thinned cortex through which the intramedullary canal was curetted and filled with artificial hydroxyapatite (Figure 3). Histological examination revealed a proliferation of cartilaginous tissues without permeative patterns or cytologic atypia, on the basis of which a pathologic diagnosis of enchondroma was made (Figure 4). The postoperative course was uneventful. No evidence of recurrence was observed at the 5-year follow-up examination.

## Discussion

Progressive diaphyseal dysplasia (PDD), or Camurati-Engelmann disease (CED), is a rare, autosomal dominant genetic disorder characterized by sclerosing bone dysplasia (3). The review by Janssens et al. of 24 CED families demonstrates that CED has worldwide penetrance (4). Clinical features of CED include leg pain, easy fatigability, and muscle weakness, which subsequently may progress to limping and a waddling gait in childhood. CED victims are usually diagnosed in childhood or in early adulthood before they reach age 30 (4). Radiographic changes include cortical thickness of the diaphysis, basilar skull sclerosis, and periosteal and endosteal sclerosis (5). However, an extensive literature review by us failed to uncover any previous reports of bone tumors (including enchondromas) associated with CED.

Enchondroma is a benign hyaline cartilage tumor that constitutes close to 20% of all cartilaginous tumors. Enchondromas most commonly present in the short tubular bones, the proximal femur, and the humerus (6). Although Milgram histologically identified cartilaginous changes around the growth plate in patients with the McCune-Albright syndrome, achondroplasia, or osteogenesis imperfecta (2), only a few published clinical case reports have described enchondromas in patients with skeletal dysplasias. Al Kaissi et al. described a clinical case of achondroplasia with an enchondroma-like multiple metaphyseal dysplasia (7). Again, to our knowledge, no association between enchondroma and CED has been reported to date.

Genetic studies of CED have discovered that CED results from a mutation in the transforming growth factor-β1 (TGF-β1) gene on chromosome 19q13.1 (8,9). TGF-β1 plays an important role in the bone remodeling process between bone formation and resorption (10,11). TGF- β1 also participates in chondrogenesis (12).

Interestingly, chromosome 19 loss has been reported as a common occurrence in both chondrosarcoma and in benign cartilaginous tumors, including enchondroma (13). Gunawan et al. reviewed 11 patients with solitary enchondromas associated with karyotypic abnormalities, one of whom also had chromosome 19 abnormalities (14). Clonal rearrangements in chromosome 19 have been reported in several cases of chondromyxoid fibroma, a relatively rare benign cartilaginous tumor (13,15). However, further studies are needed to determine the nature of the relationship between enchondroma and progressive diaphyseal dysplasia.

In conclusion, we presented here the case of an enchondroma in a 37-year-old female with Camurati-Engelmann disease. Since this is the first such report, we cannot determine whether this case was a coincidence or whether there is a genuine relationship between enchondroma and this extremely rare genetic skeletal disorder.
